# The diagnostic value of serum leptin monitoring and its correlation with tumor necrosis factor-α in critically ill patients: a prospective observational study

**DOI:** 10.1186/cc8911

**Published:** 2010-03-15

**Authors:** Ayman Abd Al-Maksoud Yousef, Yasser Mohamed Amr, Ghada Abdulmomen Suliman

**Affiliations:** 1 Department of Anesthesiology, Tanta University Hospital, El-Geish Street, Tanta, 31527, Egypt; 2 Department of Clinical pathology, Tanta University Hospital, El-Geish Street, Tanta, 31527, Egypt

## Abstract

**Introduction:**

Severe infection and sepsis are common causes of morbidity and mortality. Early diagnosis in critically ill patients is important to reduce these complications. The present study was conducted to determine the role of serum leptin at early diagnosis and differentiation between patients with manifestations of systemic inflammatory response syndrome (SIRS) and those with sepsis in patients suffering from a broad range of diseases in the intensive care unit (ICU) and its correlation with other biomarkers, such as C-reactive protein (CRP), interleukin-6 (IL-6) and tumor necrosis factor-α (TNF-α).

**Methods:**

One hundred and six adult ICU patients were observed. CRP, leptin, IL-6 and TNF-α were compared among the following groups: sepsis group (n = 40), SIRS group (n = 34) and non-SIRS group (n = 32). Patients were classified into these groups at the time of blood analysis for these biomarkers.

**Results:**

Non-significant differences were observed among patients in different groups regarding biomarkers on the day of ICU admission. On the second day of ICU admission, significant elevation of leptin, IL-6 and TNF-α occurred in the SIRS and sepsis groups. Delayed elevation of CRP started on the fourth day of ICU admission in patients with sepsis. At the end of the first week, only CRP level was elevated in septic patients.

**Conclusions:**

Serum leptin correlates well with serum level of IL-6 and TNF-α. Leptin helps to differentiate SIRS from non-SIRS patients. CRP is a classic marker of sepsis but is of late onset.

## Introduction

Severe infection and sepsis are major reasons for intensive care unit (ICU) admission and leading causes for mortality in non-coronary ICUs [1]. Infections and sepsis are accompanied by clinical and laboratory signs such as changes in body temperature, leucocytosis, and tachycardia. However, these signs of systemic inflammation may have infectious or non-infectious etiologies and are neither specific nor sensitive for sepsis [2]. Fever and leucocytosis, the classical markers of infection, have only moderate sensitivity and specificity. Fever was absent in 55% of cases of peritoneal infection while leucocytosis was absent in 35%. Early markers of septic complication would be useful for the diagnosis and treatment of sepsis [3]. C-reactive protein (CRP) has been used to follow septic patients but is a poor diagnostic and prognostic indicator because of the time taken to produce a reaction and the duration of the increase in serum concentration [4].

The systemic release of inflammatory cytokines occurs several hours earlier than the release of other markers of systemic inflammation such as acute phase protein and leucocytosis, suggesting their potential importance as diagnostic parameters in systemic inflammatory response syndrome (SIRS) and post-surgery sepsis [5]. Although cytokines such as interleukin-6 (IL-6) have been shown to relate to the severity of sepsis and patients outcome, they are not established tools for diagnosis and clinical decision making. However, IL-6 is considered a good independent early marker of postoperative sepsis, severe sepsis and septic shock [6]. Many published works have focused on the role of soluble tumor necrosis factor-α (TNF-α) as an important cytokine in inflammatory states including sepsis [7].

Leptin is an adipocyte secreted hormone. In addition to playing a role in energy regulation, leptin also regulates endocrine and immune function. It plays a role in innate and acquired immunity. Both the structure of leptin and that of its receptor suggest that leptin can be classified as a cytokine [8]. The present study was conducted to determine the role of serum leptin at early diagnosis and differentiation between patients with manifestations of SIRS and those with sepsis in patients suffering from a broad range of diseases in ICU and its correlation with other biomarkers.

## Materials and methods

After the study was approved by an investigational review board, an informed consent was obtained from patients participating in the study or from their relatives. The study was conducted over a period of nine months in the ICU of Emergency Hospital of Tanta University, Tanta, Egypt, which is a 25-bed medical/surgical ICU. One hundred and six adult ICU patients were observed. CRP, leptin, IL-6 and TNF-α were compared among the following groups: sepsis group (n = 40), systemic inflammatory response syndrome (SIRS) group (n = 34) and non-systemic inflammatory response syndrome (non-SIRS) group (n = 32), to act as a control or reference group. Patients were classified into these groups at the time of the first blood analysis for these biomarkers at ICU admission. All patients staying for more than 24 hours in the ICU were consecutively enrolled in the study. Patients who had received anti-inflammatory drugs or corticosteroids before admission, who had immunosuppressive illness, who had chronic organ failure, who had received massive blood transfusion, or whose anticipated duration of stay was under 24 hours were excluded from the study. At admission, the patient's age, sex, height and weight were recorded. Also, data were collected in the second, third and fourth days of ICU stay, then weekly, and on the day of discharge. These data include the following: clinical status: sequential organ failure assessment (SOFA) score; temperature; heart rate; respiratory rate; blood pressure; central venous pressure; laboratory analysis (complete blood count, blood urea nitrogen, blood sugar, serum sodium, potassium, calcium, aspartate aminotransferase, alanine aminotransferase, prothrombin time, albumin, CRP, leptin, IL-6 and TNF-α) and arterial blood gas analysis. Routine cultures of blood, urine and suspected sites were obtained to determine the presence of infection. We attempted to maintain the patient's hemoglobin level at 10 to 12 g/dl and central venous pressure at 8 to 12 cm H_2_O. If needed, blood products, intravascular fluid replacement and inotropic and/or vasopressor agents were administered. Each day the attending physician in the ICU evaluated all the study patients for SIRS, sepsis, severe sepsis, or septic shock. Sepsis was defined as SIRS associated with infection according to Bones' criteria [9]. The signs of SIRS were body temperature <33.6°C or >38.3°C, tachycardia (>90 beats/minute), ventilatory frequency >20 breaths/minute or PCO_2 _<32 mmHg (unless the patient was mechanically ventilated), a white cell count ≥12 × 10^9 ^litre^-1 ^or <4 × 10^9^ litre^-1 ^or >10% immature neutrophils. Severe sepsis was defined as sepsis with evidence of organ dysfunction and hypoperfusion, acute alteration of mental status, elevated plasma lactate, unexplained metabolic acidosis (arterial pH <7.3), hypoxaemia, prolonged prothrombin time or a decrease in platelet count >50% or ≤100 × 10^9^litre^1^, oliguria and hypotension defined as systolic arterial pressure <90 mmHg or a decrease of >40 mmHg. Septic shock was defined as hypotension (<90/60 mmHg) in addition to sepsis syndrome persisting despite adequate fluid resuscitation and requiring intropic support. The SOFA score is composed of scores from six organ systems (respiratory (R), cardiovascular (C), hepatic (H), coagulation (Co), renal (Re), and neurological (N)) graded from 0 to 4 according to the degree of dysfunction/failure. The aggregate score (total maximum SOFA score (TMS) is calculated, summing up the worst scores for each of the organ systems (TMS_org_) during the ICU stay [10].

### Blood sampling

Blood samples were collected in glass tubes. Blood was processed within two hours. It was centrifuged at 1,600 g for 15 minutes.

#### IL-6 and TNF-α determination using ELISA

Serum levels of IL-6 and TNF-α were determined by quantitative sandwich enzyme immunoassay (R&D Systems, Inc., Minneapolis, MN, USA) according to the manufacturer's instructions. The intensity of the colour was measured at 490 nm for both IL-6 and TNF-α.

#### Leptin determination

Serum leptin was determined by quantitative sandwich enzyme immunoassay (Ray Biotech., Inc., Minneapolis, MN, USA) according to the manufacture's instructions. The intensity of the colour was measured at 450 nm.

### Statistical analysis

Parametric data were analyzed using either ANOVA or Student's t-test while non-parametric data were analyzed using Mann-Whitney U and χ^2-^tests. Data were presented as mean and standard deviation. A *P*-value of < 0.05 was considered significant.

## Results

### Patients' characteristics

A total of 106 patients (57 men and 49 women) were included in the study. Forty patients developed septic complications during their ICU stay (sepsis group), 12 developed septic shock, 18 developed severe sepsis, and 10 patients developed sepsis without any organ dysfunction. Thirty-four patients developed manifestations of SIRS without evidence of infectious organisms (SIRS group), 10 developed non-septic complications in the form of disturbed hepatic or renal functions, electrolyte imbalance or acid-base disorders. Thirty-two medico-surgical patients showed no manifestation of SIRS (non-SIRS group). Eleven patients died, eight of whom were in septic shock and the other three were suffering from severe sepsis. There was no significant difference among the groups, except for SOFA scores at ICU admission and the duration of the stay in the ICU; SOFA scores were higher in septic patients (Table 1).

**Table 1:** Patient characteristics (mean and standard deviation)

	Sepsis group (n = 40)	SIRS group (n = 34)	Non-SIRS group (n = 32)
**Age (years**)	42 ± 10.5	46 ± 9.7	40 ± 8.2

**Sex ratio (M/F)**	21/19	18/16	18/14

**SOFA score**	11 (8 to 13)*	5 (3 to 8)	3 (2 to 5)

**Duration of ICU stay**	12.8 ± 3.6*	4.9 ± 2.2	4.4 ± 1.9

**Diagnosis**			

**Respiratory insufficiency due to:**	10	9	10
Bacterial infection.	6		
Viral infection.	4		
ARDS.		9	
COPD.			4
Bronchial asthma.			3
Pulmonary edema.			3

**Polytrauma**	9	8	7

**Orthopedic surgery**	14	11	10

**Thoracic surgery**	7	6	5

*Significant change (*P *< 0.05). M/F, male/female; ICU, intensive care unit; SIRS, systemic inflammatory response syndrome; ARDS, adult respiratory distress syndrome; COPD, chronic obstructive pulmonary disease.

The mean values of CRP at admission were 47 mg/dl in non-SIRS patients, 52 mg/dl in SIRS and 67 mg/dl in septic patients. On the second day, the mean value was 59 mg/dl in non-SIRS patients, 65 mg/dl in SIRS and 78 mg/dl in septic patients.

IL-6 mean values were nearly equal among groups and no significant differences were found between the admission values; mean IL-6 level was 7.4 pg/ml in non-SIRS, 8.5 pg/ml in SIRS and 9.6 pg/ml in septic patients, but on the second day there was a significant increase in the mean value in SIRS and septic patients: 275 pg/ml and 485 pg/ml respectively versus 21.4 pg/ml in non-SIRS patients (*P *= 0.004).

The admission mean value of TNF-α was 23.9 pg/ml in non-SIRS patients, 24.8 pg/ml in SIRS and 27.6 pg/ml in septic patients, but on the second day there was a significant increase in the mean value in SIRS and septic patients of 382 pg/ml and 407 pg/ml, respectively, versus 36 pg/ml in non-SIRS patients (*P *= 0.0032). The admission serum leptin mean values were nearly equal among patients in the different groups, they were 2.76 μg/l in non-SIRS, 2.94 μg/I in SIRS and 3.25 μg/l in septic patients, the second day levels significantly increased in septic and SIRS but not in non-SIRS patients, the mean value was 3.4 μg/l in non-SIRS compared to 30.5 μg/l in SIRS and 44.7 μg/I in septic patients (*P *= 0.005) (Table 2).

**Table 2:** Mean values of CRP, leptin, IL-6, and TNF-α levels at the second day of ICU stay

	CRP mg/dl	Leptin μg/l	IL-6 pg/ml	TNF-α pg/ml
**Non-SIRS group**	59	3.4	21.4	36

**SIRS group**	65	30.5*	275*	382*

**Sepsis group**	78	44.7*	485*	407*

*Significant change (*P *< 0.05). CRP, c - reactive protein; IL-6, interleukin-6; TNF-α, tumor necrosis factor-alpha; ICU, intensive care unit.

**Figure 1:**
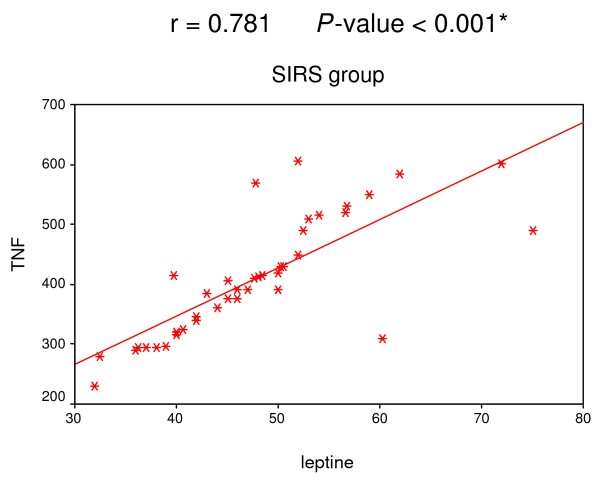
**Correlation between leptin and TNF-α in SIRS group**. Significant positive correlation between leptin and TNF-α in SIRS group.

**Figure 2:**
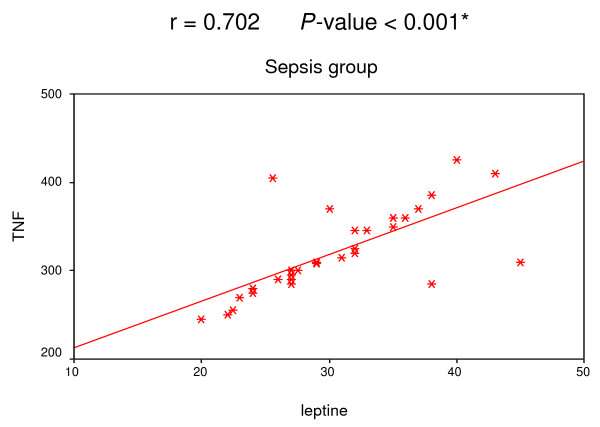
**Correlation between leptin and TNF-α in sepsis group**. Significant positive correlation between leptin and TNF-α in sepsis group.

**Figure 3:**
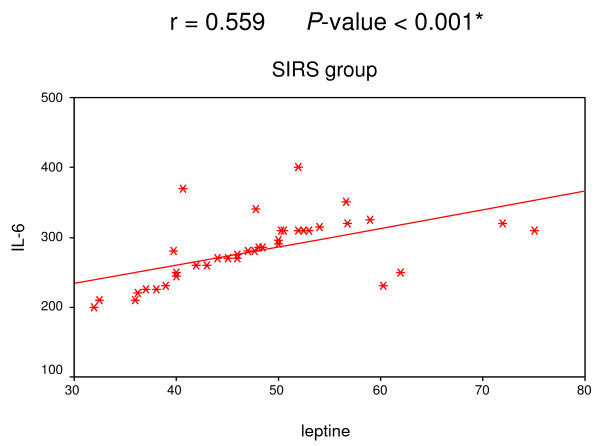
**Correlation between leptin and IL-6 in SIRS group**. Significant positive correlation between leptin and IL-6 in SIRS group.

**Figure 4:**
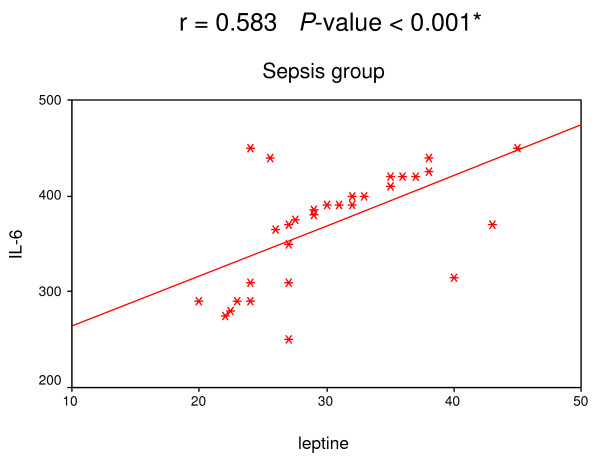
**Correlation between leptin and IL-6 in sepsis group**. Significant positive correlation between leptin and IL-6 in sepsis group.

A positive correlation was found among leptin, IL-6 and TNF-α in both SIRS and sepsis groups (Figures 1, 2, 3 and 4).

On the fourth day of the ICU stay, a significant elevation of mean value of CRP occurred in septic patients. The mean value of serum leptin, IL-6 and TNF-α declined in SIRS and septic patients but was still significantly elevated (Table 3).

**Table 3:** Mean values of CRP, leptin, IL-6 and, TNF-α levels at the fourth day of ICU stay

	CRP mg/dl	Leptin μg/l	IL-6 pg/ml	TNF-α pg/ml
**Non-SIRS group**	62	4.2	24.5	42.7

**SIRS group**	70	16.9*	184*	164*

**Sepsis group**	196*	18.6*	204*	179*

*Significant change (*P *< 0.05). CRP, c - reactive protein; IL-6, interleukin-6; TNF-α, tumor necrosis factor-alpha; ICU, intensive care unit.

At the end of the first week of the ICU stay, there was only significant elevation of mean value of CRP in septic patients. There was no significant change in the mean value of serum leptin, IL-6 and TNF-α among the different groups (Table 4).

**Table 4:** Mean value of CRP, leptin, IL-6 and TNF-α levels at the end of the first week of ICU stay

	CRP mg/dl	Leptin μg/l	IL-6 pg/ml	TNF-α pg/ml
**Non-SIRS group**	56	4.6	23.9	47.8

**SIRS group**	64	4.9	25.8	49.5

**Sepsis group**	162*	5.3	26.2	53.4

*Significant change (*P *< 0.05). CRP, c - reactive protein; IL-6, interleukin-6; TNF-α, tumor necrosis factor-alpha; ICU, intensive care unit.

**Figure 5:**
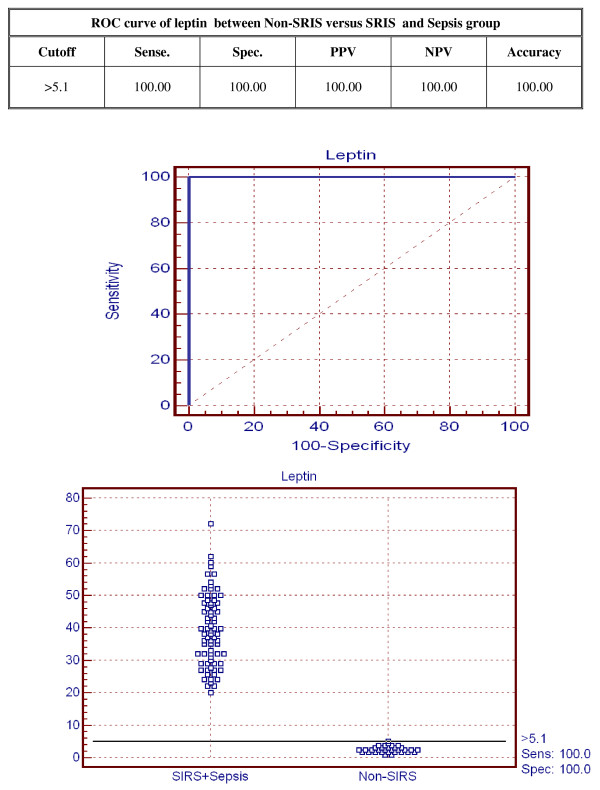
**Receiver operator curve of serum leptin between non-SIRS versus SIRS and sepsis groups**.

**Figure 6:**
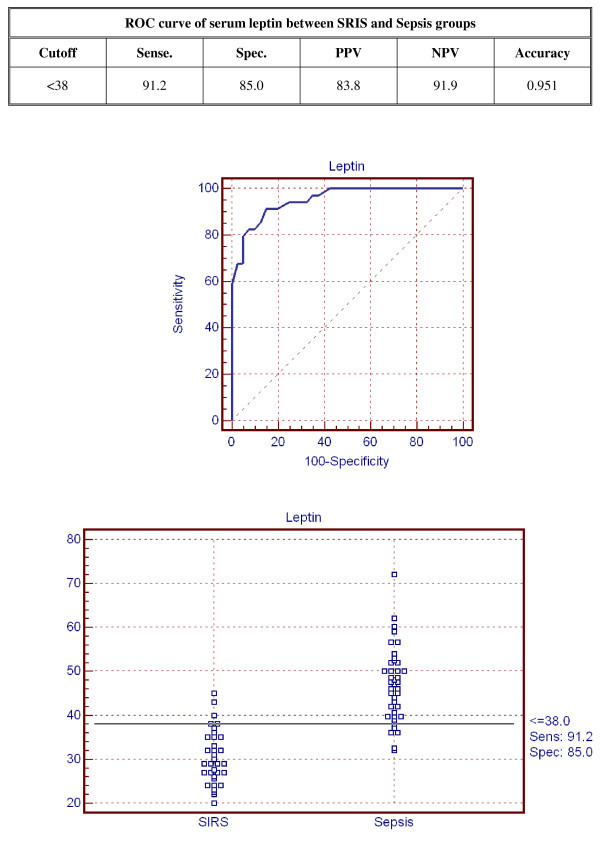
**Receiver operator curve of serum leptin between SIRS and sepsis groups**.

The accuracy of serum leptin in distinguishing non-SIRS patients from SIRS and septic patients is shown in Figure 5. A cut-off point set at 5.1 μg/l leptin had a sensitivity of 100% and specificity of 100%. The accuracy of serum leptin in distinguishing SIRS patients from septic patients is shown in Figure 6. A cut-off point set at 38 μg/l leptin gives a sensitivity of 91.2% and a specificity of 85%.

## Discussion

Prompt diagnosis and treatment with appropriate anti-microbial chemotherapy is of the utmost importance in reducing the morbidity and mortality associated with sepsis. The lack of specific early markers of infection may be responsible in part for withholding, delaying or using unnecessary antimicrobial treatment in critically ill patients. Thus, there is a need for laboratory tools that can distinguish between SIRS and sepsis [11]. In 20 to 30% of patients, the infection site is never identified. Neither imaging studies nor blood culture analysis can rule out the presence of infection. Moreover, there are classes of patients with unconfirmed infection, or for whom cultures are negative, yet they develop similar symptoms [12].

Concentrations of CRP have been used by doctors to follow septic patients, but these concentrations did not predict the outcome of disease and severity. The use of CRP concentrations has failed to allow immediate diagnosis and prognosis because of the time taken to produce a reaction and the duration of increased serum concentration. These facts may explain the lower sensitivity of CRP in the early postoperative period [13]. Povoa et al. [14] concluded that daily CRP determination could be useful as a marker of the prediction of infection. Both temperature and white cell count were not very useful in the clinical decision making process. A criticism of the work is the lack of monitoring of the other inflammatory bio-markers which would be more useful if they were combined with serial CRP determination. It is known that the persistence of TNF-α [15] and IL-6 in the serum peak levels of cytokines reveals the onset of sepsis and predicts poor outcome in septic patients [16]. Leptin is involved in the network of inflammatory mediators and during SIRS its plasma concentration increases by the action of these inflammatory mediators [17]. During a non-infectious stress response, leptin is an acute phase reactant. Studies by Maruna et al., [18] and Yamaguchi et al., [19] demonstrated that a significant correlation between leptin and TNF-alpha can be a crucial regulator of leptin generation. It is possible that pro-inflammatory cytokines induces an obesity gene (OB) transcription *in vivo *through secondary mediators such as transforming growth factor-β [20]. However, Chachkhiani et al. [5] observed that during the first 24 hours after colonic resection there is a significant increase in serum IL-6 which declined during the first 48 to 72 hours. Serum TNF-α was highest 18 to 24 hours after surgery and there was a significant elevation of plasma leptin concentration 24 hours postoperative, which rapidly returned to preoperative value 48 to 72 hours later. The concentration of leptin fails to differentiate the onset of sepsis from a non-complicated course. Bornstein et al. [21] found that the mean plasma leptin levels were three-fold higher in critically ill septic patients than healthy control adults and concluded that leptin is a stress related hormone and its role in sepsis represents an acute stress mediated response which participates in the sickness syndrome.

## Conclusions

Serum leptin increases in SIRS and sepsis and is strongly related to circulating levels of TNF-α, IL-6. Serum leptin is a powerful biomarker of SIRS patients with or without infection.

## Key messages

• Early diagnosis and differentiation of critically ill patients is crucial for better prognosis.

• Differentiation of sepsis is of utmost importance for early direction of proper anti-microbial therapy.

• Inflammatory mediators monitored in critically ill patients such as TNF-α and IL-6 have moderate efficacy and specificity for differentiation of critically ill patients.

• A positive correlation between leptin and inflammatory mediator TNF-α and IL-6 is proven in critically ill septic patients.

• Leptin monitoring is associated with a high degree of efficacy and specificity for differentiation of sepsis.

## Abbreviations

CRP: C-reactive protein; ICU: intensive care unit; IL-6: Interleukin-6; SIRS: systemic inflammatory response syndrome; SOFA: sequential organ failure assessment; TMS: total maximum SOFA score; TNF-α: Tumor necrosis factor-alpha.

## Competing interests

The authors declare that they have no competing interests.

## Authors' contributions

AAY prepared the manuscript, the statistical analysis and the patients' follow up. YMA helped in the statistical analysis and patients' follow up. GAS prepared the lab results.
